# The Impact of the COVID-19 Pandemic on Male Strength Athletes Who Use Non-prescribed Anabolic-Androgenic Steroids

**DOI:** 10.3389/fpsyt.2021.636706

**Published:** 2021-03-22

**Authors:** Barnaby N. Zoob Carter, Ian D. Boardley, Katinka van de Ven

**Affiliations:** ^1^School of Sport, Exercise and Rehabilitation Sciences, University of Birmingham, Birmingham, United Kingdom; ^2^Centre for Rural Criminology, School of Humanities, Arts, and Social Sciences, University of New England, Armidale, NSW, Australia; ^3^Drug Policy Modelling Program, Social Policy Research Centre, University of New South Wales, Sydney, NSW, Australia; ^4^Human Enhancement Drugs Network, University of New England, Armidale, NSW, Australia

**Keywords:** COVID-19, strength athletes, anabolic-androgenic steroids, mental health, exercise

## Abstract

**Background:** One sub-population potentially affected by the COVID-19 pandemic are strength athletes who use anabolic-androgenic steroids (AAS). We examined links between disruption in AAS use and training due to the pandemic and mental health outcomes in this population, hypothesising: (a) the pandemic would be linked with reduced training and AAS use; and (b) athletes perceiving greater impact on their training and AAS use would report increases in detrimental mental health outcomes.

**Methods:** Male strength athletes using AAS (*N* = 237) from 42 countries completed an online questionnaire in May 2020. A sub-sample (*N* = 90) from 20 countries participated again 4 months later. The questionnaire assessed pre-pandemic and current AAS use and training, alongside several mental health outcomes.

**Results:** At Time 1, most participants perceived an impact of the pandemic on AAS use (91.1%) and/or training (57.8%). Dependent *t*-tests demonstrated significant reductions in training frequency (*t* = 7.78; *p* < 0.001) and AAS dose (*t* = 6.44; *p* < 0.001) compared to pre-pandemic. Linear regression showed the impact of the pandemic on training was a significant positive predictor of excessive body checking (*B* = 0.35) and mood swings (*B* = 0.26), and AAS dose was a significant positive predictor of anxiety (*B* = 0.67), insomnia (*B* = 0.52), mood swings (*B* = 0.37). At Time 2, fewer participants perceived an impact of the pandemic on AAS use (29.9%) and/or training (66.7%) than at Time 1. Training frequency (*t* = 3.02; *p* < 0.01) and AAS dose (*t* = 2.11; *p* < 0.05) were depressed in comparison to pre-pandemic. However, AAS dose had increased compared to Time 1 (*t* = 2.11; *p* < 0.05). Linear regression showed the impact of the pandemic on training/AAS use did not significantly predict any mental-health outcomes. However, AAS dose was a significant negative predictor of depressive thoughts (*B* = −0.83) and mood swings (*B* = −2.65).

**Conclusion:** Our findings showed impact of the pandemic on the training and AAS use, reflected in reduced training frequency and AAS dose. However, whilst we detected some short-term consequential effects on mental health, these did not appear to be long-lasting.

## Introduction

Originating in Wuhan, China, the outbreak of the SARS-CoV-2 virus of 2019 (hereafter, COVID-19) rapidly evolved into a worldwide pandemic (1), forcing many national governments to implement isolation procedures. These measures have negatively impacted many aspects of life through termination of jobs, restrictions in travel, cessation of recreational activities, and producing a decline in national economies. Included in the impacts of the pandemic are disruptions in drug supply chains (2, 3) and access to training facilities [i.e., gymnasia, hereafter referred to as gyms (4, 5)]. One group at particular risk are strength athletes who use image and performance enhancing drugs (IPEDs), as the pandemic may have disrupted their ability to train and access certain IPEDs, potentially leading to detrimental mental health outcomes. Thus, the overarching aim of this research was to investigate whether the COVID-19 pandemic has disrupted the drug use and training behaviours of strength athletes who use IPEDs, and whether such disruption was linked with detrimental mental health outcomes.

To curb the spread of the pandemic many countries adopted strategies of social distancing and self-isolation as part of national lockdown procedures (6, 7). These strategies included the closure of gyms, thus hampering leisure and social activities. Disruption of social habits through isolation procedures has been demonstrated to negatively impact the psychological state of individuals (8–10), potentially exerting long-term detrimental psychological effects (11). Research during the COVID-19 pandemic has linked extended periods of self-isolation with confusion, anxiety, insomnia, depression, and post-traumatic stress disorder (12–16).

It is known that a sub-population of strength athletes utilise IPEDs to aid in achieving their performance- and aesthetic-based goals (17–19). Presently we focus specifically on strength athletes who use anabolic-androgenic steroids (AAS), a sub-category of IPEDs, due to the relative prevalence of AAS use amongst the range of IPEDs used by male strength athletes (20). Anabolic-androgenic steroids (AAS) are a family of chemical derivatives of the male hormone testosterone, typically taken in cycles that extend over periods of 8–16 weeks interspersed with drug free intervals (21, 22). However, research has identified presence of an AAS dependency syndrome (20), whereby AAS are administered in an almost unbroken manner despite developing adverse physical and psychological effects (23, 24). Motivations for AAS use include increasing strength, enhancing user's aesthetics, and improving performance (18, 25, 26), achieved by combining supraphysiological doses of AAS with adequate diet and training protocols (27, 28). Due to the illicit nature of AAS, purchase without a prescription may occur via several means, including buying from personal contacts and over the internet from online stores (29–33).

Anabolic compounds used in the manufacture of AAS are distributed from countries that have been heavily impacted by the COVID-19 pandemic, including China and India (34, 35), meaning that disruption in the AAS supply chain is therefore highly likely. In turn, disruptions in AAS supply may alter AAS procurement and patterns of use, forcing some athletes to prematurely terminate AAS use, potentially increasing the likelihood of developing mental health issues associated with AAS withdrawal [e.g., depressive mood, fatigue, sleep disturbances, and loss of libido (22, 36)]. Those particularly at risk from psychiatric effects are AAS dependent athletes, who have been noted to administer AAS to self-medicate withdrawal symptoms (37). Researchers have begun to explore the psychological impact of the pandemic on mental well-being (38–40). However, there is a dearth in such research with strength athletes' who use AAS.

One strategy often advocated to prevent and/or treat mental health issues is physical exercise (5, 41, 42). Research has demonstrated how exercise can alleviate symptoms of depression, anxiety, and post-traumatic stress disorder (43–45). As such, physical exercise has been encouraged to counteract the adverse physical and psychological consequences of the pandemic (46). Further, research has shown that maintenance of sport-specific fitness may be achievable for team and multidisciplinary athletes through cardiovascular based training (47), despite the COVID-19 restrictions on physical activity. However, cardiovascular training is not a viable alternative to resistance training for strength athletes (e.g., bodybuilders and weightlifters) who primarily focus on developing strength and muscle mass, as high volumes of aerobic based training can negatively affect muscle mass and hypertrophy (48–51). Lockdown protocols have seen the closure of gyms affecting professional and recreational athletes alike through disruption to training (52, 53). Strength athletes have been particularly affected by gym closures, as they require access to specialist resistance training equipment usually only available in gyms (54). Disruptions in training, therefore, present a fundamental challenge for strength athletes, further evidenced by studies showing how the inability to train effectively and access associated social support can lead to emotional distress and psychological disorders amongst athletes (55).

One psychological issue potentially affected by the pandemic is muscle dysmorphia, classified as a fixation with muscle, whereby individuals believe themselves to be inadequately small and weak, when in fact they possess a heavily muscled body. This condition elicits an obsession with exercise and intense anxiety associated with body image (56). Muscle dysmorphia is overrepresented amongst strength athletes (57, 58), and disruptions in the ability to train effectively may exacerbate psychological symptoms associated with it. To date, researchers have not examined whether psychological issues associated with muscle dysmorphia have been accentuated by the pandemic.

Based upon the arguments made to this point, through this research we sought to further our understanding on how changes in AAS use and reduced access to training facilities due to the pandemic have impacted strength athletes who use AAS. Specifically, we aimed to (a) assess the impact of COVID-19 on strength athletes' AAS use and training and (b) explore whether any disruptions in AAS use and training were linked with mental health outcomes. Based on the reviewed literature, we hypothesised the COVID-19 pandemic would have a considerable impact on athletes' use of AAS (H1), and that those who felt the pandemic had a greater impact on their use would present with greater adverse psychological effects (H2). Further, we hypothesised the pandemic would have a considerable impact on strength athletes' training (H3), and those who felt the pandemic had a higher impact on their training would present more adverse psychological issues (H4).

## Methods

### Participants

Participants at time point 1 (T1) were male strength athletes who used AAS (*N* = 237), originating from 42 countries (*n*_USA_ = 107; *n*_UK_ = 47; *n*_Canada_ = 19). They were predominantly 21–30 years of age (62.4%), single (44.7%), heterosexual (92.8%), and full-time employed or in furlough (59.9%; see Table 1). Time point 2 (T2) was a sub-sample of T1 participants (*N* = 90), originating from 20 countries (*n*_USA_ = 41; *n*_UK_ = 17; *n*_Canada_ = 6). Athletes were 21–30 years of age (66.7%), single (46.7%), heterosexual (93.3%), and full-time employed or in furlough (56.7%; see Table 1).

**Table 1:** Frequencies of participants' self-reported demographics for participants at Time 1 and Time 2.

	**T1**			**T2**		
	**Frequency**	**Percent**	**Mean ± Standard Deviation**	**Frequency**	**Percent**	**Mean ± Standard Deviation**
**Age range (years of age)**						
18–20	22	9.3		6	6.7	
21–25	79	33.3		26	28.9	
26–30	69	29.1		34	37.8	
31–35	33	13.9		13	14.5	
36–40	17	7.2		4	4.4	
41–45	7	3		4	4.4	
46–50	4	1.7		1	1.1	
51–55	3	1.3		0	0	
>55	3	1.2		2	2.2	
**Sexual orientation**						
Heterosexual	220	92.8		84	93.3	
LGBTQ+	16	6.8		6	6.7	
Prefer not to say	1	0.4		0	0	
**Marital status**						
Single	106	44.7		42	46.7	
Relationship	88	37.1		28	31.1	
Married	40	16.9		18	20	
Divorced	3	1.3		2	2.2	
**Work status**						
No income	5	2.1		2	2.2	
Temporary benefit	7	3		2	2.2	
Student	56	23.6		25	27.8	
Pension	2	0.8		0	0	
Dependent	1	0.4		0	0	
Part-time	15	6.3		6	6.7	
Full-time (in furlough)	142	59.9		51	56.7	
Self-employed	6	2.6		3	3.3	
Prefer not to say	3	1.3		1	1.1	
**Participant's average age of first use (years of age)**			24.5 ± 6.3			25.2 ± 6.9
15–20	63	26.6		21	23.3	
21–25	102	43.0		40	44.4	
26–30	40	16.9		14	15.7	
31–35	18	7.6		9	10.0	
36–40	8	3.4		3	3.3	
>40	6	2.5		3	3.3	
**Total number of cycles**			4.5 ± 4.6			4.4 ± 4.0
0	2	0.8		0	0.0	
1	44	18.6		16	17.8	
2	53	22.4		19	21.1	
3	36	15.2		15	16.7	
4	26	11.0		11	12.2	
5	18	7.6		9	10.0	
6	11	4.6		5	5.6	
≥7	47	19.8		15	16.7	
**AAS cycles in last 12 months**			1.6 ± 0.9			1.7 ± 0.7
0	9	3.8		2	2.2	
1	110	46.4		38	42.2	
2	91	38.4		38	42.2	
3	23	9.7		11	12.2	
≥4	4	1.7		1	1.2	
**Status of AAS use**						
On-cycle	50	21.1		27	30	
Off-cycle	31	13.1		13	14.4	
Blasting	42	17.7		19	21.1	
Cruising	73	30.8		21	23.3	
TRT	41	17.3		10	11.11	

*Table includes all participants at T1 (n = 237) and the participants who completed at T2 (n = 90)*.

### Measures

Data on use of IPEDs were collated at each time point. Status of use was determined at each time point by items enquiring if participants were presently “on-cycle,” “off-cycle,” “blasting,” “cruising,” or on “testosterone replacement therapy (TRT).” Weekly doses of AAS were self-reported before the onset of COVID-19 (i.e., “Prior to the COVID-19 lockdown, what was your weekly average dose of anabolic steroids?”) and at the time of the data collection (i.e., “Please indicate what estimated combined weekly dosage of anabolic steroid/s you are currently using”). Response options included “Nothing (i.e. off-cycle),” “ <300 mg,” “300–500 mg,” “501–1,000 mg,” “1,000–2,000 mg,” and “Over 2 g per week.” Ranges of AAS doses were based upon literature on therapeutic doses (59), findings from a recent literature review (20), and primary research papers (60–64), indicating current understanding of low (i.e., clinical doses <300 mg per week) and high doses (>2,000 mg per week) of AAS.

To determine the impact of the pandemic on the use of AAS, participants were asked to self-report the impact of COVID-19 on their current use of AAS (i.e., “To what degree would you rate the impact of COVID-19 on your current use of anabolic steroids?”), using a 7-point Likert scale anchored at 1 (*No Impact*) and 7 (*Extremely High Impact*). Participants were then presented with a list of different AAS and other IPEDs, and asked to identify which compounds they were currently using (e.g., ancillary drugs, peptide hormones, selective androgen receptor modulators, etc.). The AAS and IPEDs listed were based upon the extant literature [i.e., (23, 61, 65–67)].

The self-reported detrimental effects associated with AAS use were also examined at T1 and T2. Items examined psychological effects resulting from AAS use currently being experienced by participants (i.e., “Are you currently experiencing any of these effects associated with the use of anabolic steroids?”). Psychological effects included depressive thoughts, excessive body checking, increased anxiety, insomnia, and mood disturbance. These effects were based upon those associated with AAS use within the present literature (20, 26, 61, 67, 68). Items were self-reported dichotomously via “Yes” and “No” responses.

Frequency of training at T1 and T2 was self-reported (i.e., “Currently, how often do you train?”). At T1, we also asked participants to report their average training frequency in the 3 months prior to the pandemic (i.e., “Prior to the COVID-19 lockdown, how often did you train?”). Response options for training frequency ranged from 1 (*Not training*) to 6 (*More than seven times per week*). Training frequency items were derived from relevant literature [i.e., (49, 50)]. Participants were also asked to self-report the impact of COVID-19 on their training at T1 and T2 (i.e., “To what degree would you rate the impact of COVID-19 on your current training?”), using a 7-point Likert scale anchored at 1 (*No Impact*) and 7 (*Extremely High Impact*).

### Procedures

Data collections occurred at two time points during the COVID-19 pandemic. T1 occurred in April–May 2020, followed 4 months later by T2 in September–October 2020. Participants were required to be male, over the age of 18 and have taken AAS in the last 12 months prior to T1. Full ethical approval was obtained from the University of Birmingham Ethics Committee (ERN_19-1955). Participants were recruited through advertisements on bodybuilding and strength training forums where the use of IPEDs such as AAS is regularly discussed. Interested respondents were provided with a brief description of the study and a hyperlink to access the survey. Once accessed, participants were presented with an information sheet, general data protection regulation information and a consent form. Written informed consent was obtained from all participants at each time point. Participants were informed that their participation would remain entirely confidential throughout and following the study. Email addresses were required for follow-up contact at T2, and to provide successful participants with Amazon vouchers from the prize draw (see below). At the end of the T1 survey, participants were informed they would be contacted through their provided email address when it was time to complete the T2 survey in 4 months' time. Participants who completed the survey at both time points were entered into a prize draw to win a £25, £50, or £100 Amazon voucher. T1 took approximately 15 min to complete, T2 took approximately 10 min to complete.

## Results

### Descriptive Statistics

Participants reported age of first use, total number of AAS cycles and number of AAS cycles in the last 12-months for both T1 and T2 samples (Table 1). Almost all (99.2%) participants reported training regularly pre-COVID-19, with participants training predominantly ranging between four to five times per week (49.8%; see Table 2). Pre-COVID-19 weekly doses of AAS were mostly distributed between 300 and 1,000 mg per week (65.4%; see Table 2).

**Table 2:** Self-reported weekly frequencies of training and doses of AAS, impact of the pandemic on training, AAS use and psychological effects at Time 1 and Time 2.

	**Pre-COVID**		**T1**		**T2**	
	**Frequency**	**Percent**	**Frequency**	**Percent**	**Frequency**	**Percent**
**Training frequency**						
Not training	0	0	19	8	5	5.5
Once per week	2	0.8	11	4.6	0	0
2–3 times per week	12	5.1	43	18.1	10	11.1
4–5 times per week	118	49.8	85	35.9	44	48.9
6–7 times per week	97	40.9	71	30	25	27.8
>7 times per week	8	3.4	8	3.4	6	6.7
**Weekly dose of AAS**						
Not using	0	0	25	10.6	13	14.5
<300 mg per week	56	23.6	97	40.9	30	33.3
300–500 mg per week	77	32.5	42	17.7	10	11.1
501–1,000 mg per week	78	32.9	56	23.6	15	16.7
>1,000 mg per week	26	11	17	7.2	22	24.4
**Impact of COVID on training**						
No impact			21	8.9	30	33.3
Slight impact			36	15.2	26	28.9
Mild impact			23	9.7	11	12.2
Moderate impact			42	17.7	11	12.2
High impact			32	13.5	4	4.4
Very high impact			31	13.1	3	3.3
Extremely high impact			52	21.9	5	5.6
**Impact of COVID on AAS**						
No impact			100	42.2	63	70
Slight impact			26	11	6	6.7
Mild impact			19	8	5	5.6
Moderate impact			28	11.8	8	8.9
High impact			21	8.9	3	3.3
Very high impact			16	6.8	2	2.2
Extremely high impact			27	11.4	3	3.3
**Psychological effects**						
Depressive thoughts			15	6.3	9	10.0
Excess body checking			37	15.6	8	8.9
Increased anxiety			15	6.3	7	7.8
Insomnia			31	13.1	10	11.1
Mood swings			31	13.1	6	6.7

*Table includes all participants at T1 (n = 237) and the participants who completed at T2 (n = 90)*.

T1 saw slightly lower frequencies of participants still training regularly (87.3%), training mostly occurred between four to seven sessions per week (65.9%; see Table 2). 86.9% of participants reported using AAS at T1, with participants primarily indicating cruising (30.8%; see Table 1). Strength athletes mostly reported weekly doses being <300 mg per week (40.9%; see Table 2). Almost a third (32.9%) of participants self-reported experiencing one to four psychological effects, with excessive body checking being the most frequently reported (15.6%; see Table 2). Chi-square analyses identified no significant associations between off-cycle status and any psychological effects [depressive thoughts (*X*^2^ = 0.00, *p* > 0.05), excess body checking (*X*^2^ = 0.95, *p* > 0.05), increased anxiety (*X*^2^ = 0.58, *p* > 0.05), insomnia (*X*^2^ =1.38, *p* > 0.05), or mood swings (*X*^2^ = 0.36, *p* > 0.05)].

T2 indicated most participants still trained regularly (94.4%), training remained cantered between four to seven sessions per week (76.7%; see Table 2). The majority (85.6%) of participants reported using AAS at T2, with participants indicating being on-cycle (30.0%; see Table 1). Reported weekly doses mainly ranged between <300 and 500 mg (44.4%; see Table 2). Just under a quarter (22.2%) of participants reported experiencing one to five effects, with insomnia being the most frequently reported (11.1%; see Table 2). Chi square analyses identified significant associations between being off-cycle and depressive thoughts (*X*^2^ = 13.67, *p* < 0.001), increased anxiety (*X*^2^ = 4.96, *p* < 0.05), and mood swings (*X*^2^ = 14.19, *p* < 0.001).

### Impact of Pandemic on Training and AAS Use at Time 1

Most (91.1%) participants reported some impact of the pandemic on their current training, with 48.5% reporting a high to extremely high impact (see Table 2). Dependent *t*-tests demonstrated significant reductions (*t* = 7.78; *p* < 0.001) in average training frequency at T1 (*M* = 3.85; *SD* = 1.23) in comparison to pre-COVID levels (*M* = 4.41; *SD* = 0.68). More than half (57.8%) of the sample reported some impact of the pandemic on their AAS use, with 27.1% reporting a high to an extremely high impact (see Table 2). Dependent *t*-tests demonstrated significant reductions (*t* = 6.44; *p* < 0.001) in average AAS dose at T1 (*M* = 2.76; *SD* = 1.14) in comparison to pre-COVID levels (*M* = 3.31; *SD* = 0.95).

To examine whether the impact of the pandemic on training and AAS use at T1 predicted mental health outcomes at this time point, we conducted a series of hierarchical logistic regression analyses (see Table 3). In each of these analyses we entered T1 training frequency and AAS dose in the first step to examine and control for their effects on the outcome variable, before entering the impact of the pandemic on training and AAS use at T1 in the second step. These analyses showed that at T1, AAS dose was a significant positive predictor of anxiety, insomnia, and mood disturbance, and the impact of the COVID-19 pandemic on training was a significant positive predictor of excessive body checking and mood disturbance when controlling for the effects of training frequency and AAS dose. There were no significant predictors of depressive thoughts.

**Table 3:** Logistic regression of mental health outcomes on impact of the pandemic on training and AAS use at Time 1.

**Variable**	***B***	***SE B***	**Wald χ^**2**^**	**Odds ratio**	***R*^**2**^**	**Model χ^**2**^**
**EXCESSIVE BODY CHECKING**						
Step 1					0.06	7.95^*^
Training frequency	0.30	0.18	2.79	1.35		
AAS dose	0.28	0.16	3.08	1.32		
Step 2					0.13	18.95^**^
Impact on training	0.35	0.12	8.21^**^	1.42		
Impact on AAS use	−0.02	0.10	0.02	0.99		
**DEPRESSIVE THOUGHTS**						
Step 1					0.04	3.71
Training frequency	−0.37	0.23	2.73	0.69		
AAS dose	0.36	0.24	2.26	1.43		
Step 2					0.05	4.61
Impact on training	0.13	0.18	0.55	1.14		
Impact on AAS use	0.02	0.14	0.03	1.02		
**ANXIETY**						
Step 1					0.09	8.15^*^
Training frequency	−0.13	0.26	0.27	0.87		
AAS dose	0.67	0.24	7.70^**^	1.94		
Step 2					0.11	10.31^*^
Impact on training	0.23	0.17	1.86	1.26		
Impact on AAS use	−0.04	0.15	0.05	0.97		
**INSOMNIA**						
Step 1					0.07	9.24^*^
Training frequency	−0.11	0.18	0.38	0.90		
AAS dose	0.52	0.17	8.86^**^	1.68		
Step 2					0.09	11.08^*^
Impact on training	−0.15	0.13	1.38	0.86		
Impact on AAS use	0.14	0.12	1.36	1.15		
**MOOD DISTURBANCE**						
Step 1					0.04	4.70
Training frequency	−0.14	0.17	0.72	0.87		
AAS Dose	0.37	0.17	4.63^*^	1.45		
Step 2					0.07	9.28
Impact on training	0.26	0.13	4.21^*^	1.30		
Impact on AAS use	−0.06	0.11	0.29	0.94		

*All dependent variables were coded 0 = No, 1 = Yes*.

* *p 0.05*,

** *p 0.01*.

### Impact of Pandemic on Training and AAS Use at Time 2

Two-thirds (66.7%) of participants reported some impact of the pandemic on their training at T2, with 13.7% reporting a high to extremely high impact (see Table 2). Dependent *t*-test analyses demonstrated that training frequency at T2 (*M* = 4.13; *SD* = 1.07) was depressed (*t* = 3.02; *p* < 0.01) in comparison to pre-COVID levels (*M* = 4.43; *SD* = 0.69). Further, although training frequency at T2 was higher than at T1 (*M* = 3.94; *SD* = 1.27), the difference was not statistically significant (*t* = 1.44; *p* > 0.05). Almost a third (29.9%) of participants reported some impact of the pandemic on their AAS use at T2, with 8.8% reporting a high to extremely high impact (see Table 2). Dependent *t*-tests demonstrated average AAS dose at T2 (*M* = 3.03; *SD* = 1.44) was significantly higher (*t* = 2.11; *p* < 0.05) than at T1 (*M* = 2.67; *SD* = 1.13), but still significantly lower (*t* = 2.11; *p* < 0.05) than the average pre-COVID dose (*M* = 3.36; *SD* = 0.94).

To examine whether the impact of the pandemic on training and AAS use at T2 predicted mental health outcomes at this time point, we conducted a series of hierarchical logistic regression analyses (see Table 4). In each of these analyses we entered T2 training frequency and AAS dose in the first step to examine and control for their effects on the outcome variable, before entering the impact of the pandemic on training and AAS use at T2 in the second step. These analyses showed that at T2, AAS dose was a significant negative predictor of mood disturbance and depressive thoughts. The impact of the COVID-19 pandemic on training and AAS did not predict any of the mental health outcomes at T2, and there were no significant predictors of excessive body checking, anxiety, and insomnia at this time point.

**Table 4:** Logistic regression of mental health outcomes on impact of the pandemic on training and AAS Use at Time 2.

**Variable**	***B***	***SE B***	**Wald χ^**2**^**	**Odds ratio**	***R*^**2**^**	**Model χ^**2**^**
**EXCESSIVE BODY CHECKING**						
Step 1					0.11	4.49
Training frequency	0.84	0.45	3.34	2.32		
AAS dose	−0.25	0.28	0.85	0.78		
Step 2					0.11	4.54
Impact on training	−0.05	0.30	0.03	0.95		
Impact on AAS use	0.07	0.31	0.06	1.08		
**DEPRESSIVE THOUGHTS**						
Step 1					0.16	7.35^*^
Training frequency	0.04	0.30	0.02	1.04		
AAS dose	−0.83	0.38	4.81^*^	0.44		
Step 2					0.17	7.65
Impact on training	0.12	0.26	0.20	1.12		
Impact on AAS use	0.02	0.27	0.00	1.02		
**ANXIETY**						
Step 1					0.02	0.82
Training frequency	−0.03	0.35	0.01	0.97		
AAS dose	−0.25	0.31	0.67	0.78		
Step 2					0.03	1.22
Impact on training	0.13	0.28	0.22	1.14		
Impact on AAS use	0.03	0.29	0.01	1.03		
**INSOMNIA**						
Step 1					0.04	1.86
Training frequency	0.47	0.37	1.58	1.60		
AAS dose	−0.15	0.24	0.35	0.87		
Step 2					0.05	2.11
Impact on training	−0.13	0.27	0.23	0.88		
Impact on AAS use	0.11	0.27	0.15	1.11		
**MOOD DISTURBANCE**						
Step 1					0.48	18.60^***^
Training frequency	0.95	0.49	3.73	2.58		
AAS dose	−2.65	1.02	6.79^**^	0.07		
Step 2					0.55	21.71^***^
Impact on training	0.38	0.42	0.82	1.46		
Impact on AAS use	0.34	0.41	0.69	1.41		

*All dependent variables were coded 0 = No, 1 = Yes*.

* *p 0.05*,

** p 0.01, and

*** *p 0.001*.

## Discussion

This study aimed to assess the impact of the COVID-19 pandemic on strength athletes' AAS use and training, and whether any impact/s on AAS use and training were linked with mental health outcomes. Our findings partly confirmed our hypotheses in that the COVID-19 pandemic demonstrated impact on the AAS use behaviours and training of strength athletes who use AAS (H1 and H3), but did not demonstrate any long-term consequential effects on mental health (H2 and H4). These findings are important, as until now there has been a dearth in research identifying just how strength athletes who use AAS have been affected by the COVID-19 pandemic.

Our findings show that at T1, 57.8% of strength athletes perceived some impact of the pandemic on their AAS use, reducing to 29.9% of participants at T2. This was reflected in average AAS dose being lower than it was pre-pandemic at both T1 and T2. However, the impact of COVID-19 on AAS did not predict any of the mental health issues under study at either time point. This may be because only around a quarter at T1 and a tenth at T2 perceived this impact to be a high impact or greater. Thus, although their AAS use was reduced, it seems on the whole the degree of impact was not sufficient to negatively impact mental health. However, our findings did illustrate that at T1, AAS dose was a significant positive predictor of anxiety, insomnia, and mood swings, meaning that individuals who took higher doses were more likely to experience these mental health issues. Although less common, it has been reported in the literature that some individuals will use non-prescribed AAS to cope with stressful circumstances (69) or anxiety (70). It therefore could be that individuals who took higher doses were more anxious and stressed about the COVID-19 pandemic and therefore took higher doses in an attempt to cope with stress they were experiencing. As such, support services for AAS users should keep in mind that an increase in an athletes' AAS dose may not always be training related, and could be associated with an increase in mental health issues.

Interestingly, when looking at T2, we see that AAS dose was a significant negative predictor of mood disturbance and depressive thoughts, such that lower doses were associated with increases in these mental health issues. This was particularly the case for those who were not using at all (i.e., off-cycle), with such athletes more likely to experience depressive thoughts, increased anxiety, and mood swings compared to those on-cycle. Although these findings contrast with the equivalent analyses at T1, they are more consistent with the extant AAS use literature, as this pattern is consistent with symptoms of AAS withdrawal. Such symptoms typically appear upon discontinuation of AAS use due to AAS-induced hypogonadism (deficiency in testosterone), especially if individuals have used AAS for prolonged periods (71, 72). The return to a more regular pattern of associations between AAS dose and mental health outcomes at T2 further reinforces the possibility that the positive associations between AAS dose and detrimental mental health outcomes at T1 represented a specific response to the COVID-19 pandemic. The links between AAS use and mental health identified here highlights the importance of people who use AAS having access to health services to obtain treatment. It is, however, well-established that access to health services for this sub-population is generally limited; not only due to the lack of treatment available (73) but also due to a lack of knowledge amongst health professionals about these substances (74, 75). This lack of access has been exacerbated due to the COVID-19 pandemic with health services, including alcohol and other drugs services, needing to close down or restricting their access (76, 77). It is therefore imperative that more is done to produce well-informed and accessible health services specific to those who use non-prescribed AAS, which can be utilised despite the presence of a global pandemic such as COVID-19.

The perceived impact of COVID-19 on training alongside subsequent reductions in training frequency comparative to pre-COVID-19, at both T1 and T2, indicate notable disruptions in the ability of strength athletes' to train effectively during the pandemic. This is concerning, as several studies in the early COVID-19 stages have shown that a reduction in physical activity has a negative impact on mental health and well-being (78, 79). Our findings likewise showed the perceived impact of the pandemic on their training was negatively linked with aspects of their psychological health at T1. Specifically, it was a significant positive predictor of excessive body checking and experiencing mood swings. Importantly, excessive body checking can be indicative of body image (e.g., muscle dysmorphia) or eating (80, 81) disorders. Considering muscle dysmorphia is not uncommon amongst strength athletes (57, 58), elevated rates of stress due to reduced training may contribute to increasing risk for developing a body image disorder. Indeed, Swami et al. (82) showed COVID-19-related stress and anxiety was associated with negative body image, and for men in particular, it was associated with greater muscularity dissatisfaction which likewise can be a sign of muscle dysmorphia. It is therefore important to better understand the impact of COVID-19, and associated factors including gym closures and disruptions in training, on body image disorder risk in strength athletes who use AAS. Such increased understanding would help inform interventions to better support this population.

Of note though, at T2 the impact of the COVID-19 pandemic on training did not predict any of the mental health outcomes. Whilst our data cannot speak to mechanistic pathways, it may be that many individuals were able to come to terms with the restrictions and adapt their training regimes and/or training goals to lessen the perceived impact of the pandemic on their training. This possibility is supported by the reduction in the number of athletes (i.e., 91.1% at T1 and 66.7% at T2) perceiving an impact of the pandemic on their training at T2 compared to T1. Especially when you consider the change in the percentage of athletes (i.e., 48.5% at T1 and 13.7% at T2) perceiving a high, very high, or extremely high impact of the pandemic on their training. However, the question remains as to how long strength athletes can continue to adapt in this way and keep up this routine to accommodate the impact the pandemic has had on their ability to train normally. Further, reduced access to the gym and associated social-support networks may lead to increased social isolation over time, which can increase psychical inactivity (83), for example, due to factors such as reduced motivation and boredom.

### Limitations and Future Recommendations

The present study was not without limitations. The study experienced a high attrition rate (62.1%) during the transition from T1 to T2 data collection, but not to a level that would render the results as non-meaningful [see (84)]. Although statistically significant results were determined at T2, the reduction in power—due to the attrition rate—reduced our ability to detect statistically significant results with weaker effect sizes in comparison to at T1. Possible explanations for this attrition rate include reminder emails being automatically redirected to spam/junk folders, participants experiencing COVID survey fatigue, participants forgetting their participation in the study, and participants having reduced motivation to continue their participation as lockdown restrictions were eased (i.e., strict restrictions may have been a primary motivator of participation for many at T1). Generalizability may also have been affected due to the openness of participants about their use of AAS. Specifically, those who are more open about their AAS use may have opted to partake in the study, with those who are not avoiding participation. Further, use of self-report items may have led to socially desirable responses and incidences of recall bias. This study was also limited due to the two time-point longitudinal design, limiting the analyses that could be conducted on the data; increasing the frequency of time-points would facilitate a design in which longitudinal relationships could be determined.

Our recommendations for future research are aimed at developing longitudinal studies to further understand the impact of COVID-19 and the risk of developing body image disorders and longitudinal investigations on the robustness of strength athletes maintaining their training through social isolation protocol. Further recommendations include the provision of position statements identifying the importance of access to adequate training facilities suitable for all exercise disciplines during pandemics, to aid in guiding governmental procedures for future lockdown protocols.

## Conclusion

Our findings support our hypotheses that the COVID-19 pandemic demonstrated impact on the training and AAS use behaviours of strength athletes who use non-prescribed AAS. Reductions in both training frequency and weekly dose of non-prescribed AAS reflected the impact of the global pandemic on the athletes' training and drug-use behaviours. However, our analyses did not support any consequential effects of the impact of COVID-19 on non-prescribed AAS use and adverse mental health outcomes. Ongoing longitudinal analyses will help determine whether more time was needed for such effects to manifest, especially if the athletes under study return to lockdown conditions when consequent impacts are heightened.

## Data Availability Statement

We will not be making the raw data available as it was not in the remit of our ethics application.

## Ethics Statement

The studies involving human participants were reviewed and approved by University of Birmingham Ethics Committee. The participants provided their written informed consent to participate in this study.

## Author Contributions

BZ and IB collaborated to develop and design the project. BZ collected the data for the project. BZ wrote the initial draft of the introduction and method sections. IB conducted the data analysis, in collaboration with BZ. KV wrote the initial draft of the discussion. All authors contributed to the article and approved the submitted version.

## Conflict of Interest

The authors declare that the research was conducted in the absence of any commercial or financial relationships that could be construed as a potential conflict of interest.
